# Ovarian Reserve and Pelvic Ultrasound Assessment in Familial Mediterranean Fever

**DOI:** 10.7759/cureus.28027

**Published:** 2022-08-15

**Authors:** Muhammed Okuyucu, Ayse Zehra Ozdemir, Demet Yalcin Kehribar, Metin Ozgen

**Affiliations:** 1 Internal Medicine, Ondokuz Mayıs University Faculty of Medicine, Samsun, TUR; 2 Obstetrics and Gynecology, Ondokuz Mayıs University Faculty of Medicine, Samsun, TUR; 3 Rheumatology, Ondokuz Mayıs University Faculty of Medicine, Samsun, TUR

**Keywords:** anti-interleukin-1 treatment, anti-müllerian hormone, ovarian reserve, familial mediterranean fever, pregnancy rate

## Abstract

Introduction

Familial Mediterranean fever (FMF) is an inflammatory rheumatic disease that affects people in their reproductive period. The aim of this study was to investigate the number of gravida, ovarian reserve, and ovarian doppler characteristics in FMF patients.

Methods

The study design is cross-sectional. Between November 1, 2018, and October 31, 2019, 40 FMF patients, and 40 age-matched volunteers were included in the study. Early follicular phase follicle-stimulating hormone (FSH), luteinizing hormone (LH), estrogen (E2), progesterone, and anti-Mullerian hormone (AMH) levels, as well as ovarian volume, antral follicle count (AFC), ovarian stromal artery doppler findings, and pelvic pathologies, were evaluated.

Results

The number of gravida, and the AFC was significantly higher in the control group (16.00 ± 5.22) compared to the patients with FMF (13.00 ± 4.09) (p = 0.026). LH values were significantly higher in the FMF group. Thirteen patients (32.5%) received anakinra and colchicine, and 27 patients (67.5%) received only colchicine. There was no significant difference between the patients receiving anakinra, and the patients receiving colchicine in terms of AMH, FSH, AFC, and E2 values.

Conclusion

FMF patients were found to have low gravida and AFC, and a significant portion was observed to have pelvic fluid and hydrosalpinx. In conclusion, the presence of pelvic fluid, hydrosalpinx, and low AFC persist in FMF patients despite colchicine and/or anti-interleukin-1 treatments. The low gravida may be related to these pathologies detected in patients with FMF.

## Introduction

Familial Mediterranean fever (FMF) is a hereditary autoinflammatory disorder characterized by attacks of fever and serosal inflammation [1]. It is most common in Turkey with a prevalence of 1/150 and 1/10.000 [2]. It severely reduces the quality of life due to chronic inflammation between attacks, and periods [3]. 

Mutations in the Mediterranean fever (MEFV) gene, which encodes the pyrin protein, are responsible for the pathogenesis of FMF [4]. The pyrin protein is secreted by neutrophils, lymphocytes, and dendritic cells and enables nuclear factor kappa-beta (NF-κB) activation to induce interleukin (IL)-1β synthesis, and apoptosis. Uncontrolled secretion of IL-1β results in neutrophil activation occurring in an uncontrolled manner, which leads to an abnormal inflammatory response [1]. Colchicine treatment prevents attacks and reduces the development of amyloidosis [5]. Anakinra is a recombinant non-glycosylated IL-1 receptor antagonist used in FMF patients who are unresponsive to colchicines [6,7].

FMF usually affects people of reproductive age. FMF and associated amyloidosis may cause both male and female infertility [8]. Increased infertility rates have been observed in FMF patients compared to the normal population, more specifically in those who were not receiving colchicine treatment. Ovulatory disorders and peritoneal adhesions have been reported in these patients [5]. Another study reported that patients who were unresponsive to colchicine were at higher risk for infertility. Studies have also indicated that FMF patients had lower ovarian reserve [4,9].

Although it is known that colchicine treatment reduces peritoneal adhesions [5], it is still controversial whether or not colchicine can reduce infertility. Theoretically, the prevention of systemic inflammatory attacks with colchicine treatment may prevent infertility in FMF patients. However, there are few studies on whether or not infertility improves after starting colchicine treatment. Furthermore, while anti-IL-1 treatment, used in FMF patients unresponsive to colchicine, has been shown to be effective in controlling FMF attacks [10], there is no study demonstrating the effects of anti-IL-1 treatment on fertility.

In this study, we aimed to evaluate the gravida and the changes in ovarian reserve and ovarian doppler findings in FMF patients receiving colchicine, and anakinra.

## Materials and methods

This study was approved by the Ondokuz Mayıs University Clinical Research Ethics Committee (Approval number: OMU KAEK 2019/314). Informed consent was obtained from all participants. This study was performed according to the principles of the Declaration of Helsinki.

The study had a cross-sectional study design. FMF patients admitted to our internal medicine department between November 1, 2018, and October 31, 2019, were included in this study. Livneh and Tel-Hashomer classification criteria were used as diagnostic criteria [11].

Inclusion criteria

Our study included 40 patients aged between 18-40 years who were diagnosed with FMF; 27 of these patients only received colchicine (1-1.5 mg daily) and 13 patients received anakinra due to non-responsiveness to colchicine. The medical records were evaluated to obtain data such as the patients' genetic mutations, drug use, and the presence of amyloidosis. Additionally, 40 age-matched women (the hospital staff) with no diseases were included as the control group.

Exclusion criteria

Patients with early menopause, infertility, any gynecological disease (polycystic ovary syndrome, abnormal uterine bleeding, and history of previous gynecological surgery), smokers, presence of other inflammatory diseases, and patients with chronic liver or kidney disease were excluded from the study. Patients using anakinra for less than one year were excluded from the study. 

Laboratory analysis

The peripheral venous blood sample taken on the second or third day of the cycle was examined for follicle-stimulating hormone (FSH), luteinizing hormone (LH), estradiol (E2), and progesterone by enzyme-linked immunosorbent assay (ELISA) method. AMH levels were measured in 5 mL venous blood drawn specifically for the present study by the ELISA method.

Ultrasound

On the same day, transabdominal pelvic ultrasound was performed to determine the antral follicle count and tubal pathology and it was calculated according to the formula of ovarian volume (length × width × height) by a gynecologist (AZO). Besides, the ovarian arteries were examined by doppler ultrasound.

Statistical analysis

The sample size was determined according to a power of at least 0.80 and a type I error of 0.05 for each variable (Benchmark Six Sigma; https://www.benchmarksixsigma.com/). The continuous (numeric) variables were presented as descriptive statistics such as median, mean, standard deviation, minimum, and maximum; whereas the categorical variables were presented as numbers and percentages. Shapiro Wilk (n < 50) was used to determine whether the continuous variables in the study were normally distributed and non-parametric tests were performed for the non-normally distributed variables. The Mann-Whitney U test was used in the comparison of the measurements between the groups. The chi-square test was used to determine the correlation between the categorical variables. The statistical significance level was accepted as 5% and the data were analyzed using the SPSS software v. 24 for Windows (IBM Corporation, Armonk, NY).

## Results

The control group had similar average age as the FMF group (28.0 ± 6.3 years (min = 18, max = 39), 27.0 ± 6.5 years (min = 19, max = 39), respectively; p = 0.638). A comparison of the hormone levels of the FMF patients and the control group is presented in Table 1. While only LH levels were significantly higher in the FMF group, there was no significant difference between the two groups in terms of other hormones.

**Table 1:** The levels of hormones in study groups. FMF: familial Mediterranean fever, FSH: follicle stimulating hormone

	Control (N = 40)	FMF (N = 40)	P-Value
FSH (IU / L)	6.55 ± 2.37	7.40 ± 4.12	0.119
Luteinizing Hormone (IU / L)	2.50 (0.72 - 9.80)	6.80 (1.20 - 20.00)	< 0.001
Estradiol (pg / ml)	33.00 (12.00 - 98.00)	37.50 (11.00 - 99.00)	0.619
Progesterone (ng / ml)	0.20 (0.10 - 0.80)	0.20 (0.10 - 7.30)	0.068
Anti Müllerian Hormone (ng / ml)	2.08 (1.05 - 4.60)	2.49 (0.13 - 4.81)	0.318

Gravida was significantly low in FMF patients, while there was no significant difference in number of abortion (Table 2). According to ultrasound findings, there was no significant difference between the two groups according to ovarian volume, PI, and RI values however, FMF patients had significantly lower antral follicle count (Table 2). In addition, in the FMF group, 16 patients had pelvic fluid (40%), five hydrosalpinx (12.5%), and one patient amyloidosis (2.5%).

**Table 2:** Gravida, abortion, ovarian reserve, and ovarian Doppler characteristics in the study groups. FMF: familial Mediterranean fever

		Healthy Control N (%)	FMF N (%)	P-Value
Age (years)		28.0 ± 6.3	27.0 ± 6.5	0.638
Gravida	0	17 (42.5%)	29 (72.5%)	0.014
	1	11 (27.5%)	2 (5.0%)	
	2	11 (27.5%)	7 (17.5%)	
	3	1 (2.5%)	2 (5.0%)	
Abortion	0	36 (90.0%)	39 (97.5%)	0.346
	1	3 (7.5%)	1 (2.5%)	
	2	1 (2.5%)	0(0.0%)	
Ovarian Volume (cm^3^) (length×width×height)		11.5 (min: 3.6 – max: 29.7)	11.0 (min: 2.7 – max: 40.6)	0.224
Antral Follicle Count		16.00 ± 5.22	13.00 ± 4.09	0.026
Pulsatility Index		2.10 ± 0.79	1.60 ± 0.97	0.096
Resistive Index		0.20 (min: 0.10 – max: 0.80)	0.11 (min: 0.3 – max: 0.80)	0.752
Pelvic Fluid (N)		-	16 (40.0%)	
Hydrosalpinx (N)		-	5 (12.5%)	

In the FMF group, anakinra 100 mg/day + colchicine 1-1.5 mg/day were used in 13 patients (32.5%), whereas only colchicine was used in 27 patients (67.5%). When FMF patients were classified into groups according to the treatment they received, there was no significant difference between the colchicine, and colchicine + anakinra according to hormone levels or ultrasound findings (Table 3). There was also no difference between these two groups according to gravida (p = 0.500).

**Table 3:** Hormone levels and ultrasound findings according to the treatments received by familial Mediterranean fever patients.

	Colchicum	Colchicum+Anakinra	P-Value
Follicle-Stimulating Hormone (IU/L)	6.30 (4.60-14.00)	7.80 (3.40-24.00)	0.409
Estradiol (pg/ml)	25.0 (13.0-88.0)	40.00 (11-99)	0.793
Anti-Müllerian Hormone (ng/ml)	2.83 (1.11-3.78)	2.06 (0.13-4.80)	0.860
Antral Follicle Count	13.00 (5.00-17.00)	13.00 (3.00-20.00)	0.568
Pulsatility Index	1.70 (1.05-4.50)	1.40 (0.90-4.20)	0.387
Resistive Index	0.14 (0.10-0.66)	0.10 (0.03-0.80)	0.375

When FMF patients with M694V mutation (n = 40 patients) were compared with FMF patients without M694V mutation (n = 15 patients), there were no differences in pregnancy rates, AMH hormone levels, and antral follicle count (p > 0.05 all).

## Discussion

This study aimed to investigate pregnancy rate, ovarian reserve, and ovarian doppler findings associated with infertility causes in FMF patients undergoing colchicine and anti-IL-1 treatment. Despite treatment, FMF patients had a significantly lower pregnancy rate compared to the control group. The most important data to explain the low fertility rate we found in FMF patients were high rates of pelvic fluid accumulation, hydrosalpinx, and low antral follicle count.

Although female FMF patients have increased infertility, its causes are unknown. It has been reported that recurrent peritonitis attacks resulting in peritoneal adhesions, ovarian amyloidosis, and tubal dysfunction may lead to infertility [12-15]. Colchicine treatment largely prevents recurrent peritonitis attacks. However, studies on the positive or negative effects of colchicine and anti-IL-1, currently used in treatment, on fertility are limited.

A study by Atas et al. which defined infertility as the inability to conceive despite 12 months of unprotected intercourse, reported an infertility rate of 14.6% among female FMF patients and that this rate was higher than the national prevalence of female infertility (8.6%) [12]. Our study was not designed and standardized to determine infertility status in FMF patients. However, female FMF patients were found to have a significantly lower pregnancy rate compared to the control group in our study. The low gravida in FMF patients may be related to their FMF-related problems.

Zayed et al. conducted a study that included 74 infertile FMF patients and reported that the causes of infertility were similar to the normal population [16]. In their study, ultrasound or laparoscopy detected pelvic fluid in 75.5% of the patients. This fluid was thought to be an aseptic inflammatory fluid that disrupted oocyte pick up. It was observed that this fluid surrounding the tubes and ovaries disrupted the relationship between sperm and oocytes. In our study, we detected pelvic fluid in 40% of FMF patients. This result demonstrates that pelvic fluid accumulation persists despite colchicine and anti-interleukin treatments.

In a study, Oner et al. compared FMF patients with a healthy control group and revealed a higher FSH level and a lower antral follicle count in FMF patients [9]. AMH levels were not evaluated in the study. Similar to the aforementioned study, we also determined that FMF patients had significantly lower antral follicle count. However, the FMF group and control group had similar AMH levels. Şahin et al. demonstrated that AMH levels in FMF patients do not differ from the normal population [4]. In our study, the comparison of FMF patients with the control group did not reveal any difference in AMH values. Although FMF patients have similar AMH levels as the normal population, lower antral follicle count in FMF patients draws attention and requires further investigation. Perhaps, similar to the euthyroid sick syndrome, systemic inflammation in FMF may reduce the effect of AMH on antral follicles.

There are some limitations to this study. Firstly, it would be better to include more number of patients. However, FMF is not a very common disease. It would be better to have the patients questioned about their sexual behaviors, recurrent peritonitis attacks, and economic status. Lastly, the time of abortion was not evaluated in this study. The study would have been more valuable had it been designed to compare infertility rates and standardized the participants accordingly.

## Conclusions

Our study is the first to evaluate the effects of anti-IL-1 treatments on infertility and ovarian reserve. Furthermore, we believe our study is valuable, as it evaluates both gravida and ovarian reserve within the same FMF cohort. Therefore, low gravida rate and presence of pelvic fluid, and low antral follicle count, which we believe are potential causes, have been demonstrated. In conclusion, the presence of pelvic fluid and low antral follicle count persist in FMF patients despite colchicine and anti-IL-1 treatments, therefore pregnancy rate is lower compared to the normal population.
